# SP1-induced HOXD-AS1 promotes malignant progression of cholangiocarcinoma by regulating miR-520c-3p/MYCN

**DOI:** 10.18632/aging.103660

**Published:** 2020-08-28

**Authors:** Jinglin Li, Xingming Jiang, Zhenglong Li, Lining Huang, Daolin Ji, Liang Yu, Yongxu Zhou, Yunfu Cui

**Affiliations:** 1 Department of General Surgery, The 2nd Affiliated Hospital of Harbin Medical University, Harbin 150086, Heilongjiang, China

**Keywords:** cholangiocarcinoma, prognosis, HOXD-AS1, miR-520c-3p, MYCN

## Abstract

The purpose of this article is to explore the function and mechanism of HOXD-AS1 in cholangiocarcinoma. TCGA, StarBase and JASPAR were applied to predict the differential expression and molecular mechanism. The qRT-PCR was conducted to detect molecular expression. The effect of HOXD-AS1 on tumor proliferation, metastasis and stemness was measured through corresponding experiments. ChIP, luciferase reporter and RIP assays were implemented to explore the regulatory mechanism of HOXD-AS1 in CCA. In this study, HOXD-AS1 expression was significantly upregulated in CCA tissues and cells compared with control groups, respectively. Increased HOXD-AS1 was markedly correlated with lymph node invasion, advanced TNM stage and poor survival of CCA patients. Moreover, HOXD-AS1 was confirmed to be an unfavorable independent prognostic factor for CCA patients. Functionally, gain- and loss-of-function experiments demonstrated that HOXD-AS1 facilitated tumor proliferation, migration, invasion, EMT, stemness and drug resistance *in vitro* and *in vivo*. For the mechanism, transcription factor SP1-induced HOXD-AS1 upregulated oncogene MYCN through competitively binding to miR-520c-3p. Furthermore, HOXD-AS1-induced malignant phenotypes were rescued by interfering miR-520c-3p and MYCN, respectively. SP1/HOXD-AS1/miR-520c-3p/MYCN plays a vital role in initiation and progression of CCA, and HOXD-AS1 is expected to be an efficient biomarker and therapeutic target.

## INTRODUCTION

Cholangiocarcinoma (CCA) is a type of malignant tumor of digestive system that originates from bile duct epithelial cells [1]. Due to highly malignant biological behavior, the prognosis of CCA patients is extremely poor. Radical excision is the only effective way to cure CCA. However, the majority of patients have reached an advanced stage by the time of diagnosis, resulting in losing the opportunity for radical surgery [2]. Improving the prognosis of CCA patients needs to be considered from the direction of early diagnosis and non-surgical treatment. Therefore, exploring key regulatory pathways involved in the development of CCA is urgently needed. The aim is to obtain valuable early diagnostic biomarkers and therapeutic targets.

Long noncoding RNAs (lncRNAs) are a group of non-protein-coding RNA with longer than 200 nucleotides. Because there is no obvious open reading frame, lncRNAs have no or limited protein-coding capability [3]. LncRNAs have been verified to exhibit a crucial regulatory role in many diseases, especially in the occurrence and development of tumors [4]. They can regulate target genes at epigenetic, transcriptional and post-transcriptional levels via acting as competitive endogenous RNA (ceRNA), transcript guide, regulatory signal, transcript decoy, and protein scaffold [5, 6]. Among these tumor-related lncRNAs, HOXD cluster antisense RNA 1 (HOXD-AS1), also known as HOXD antisense growth-associated long non-coding RNA (HAGLR), has been affirmed to function as an oncogene in diverse digestive system tumors, such as gastric cancer [7], hepatocellular carcinoma [8], and colorectal carcinoma [9]. HOXD-AS1 originates from the homeobox gene family, and this family plays a vital role in embryogenesis and organogenesis. Homeobox dysregulation is involved in developmental deficiency of human body [10]. Many ncRNAs are recognized in human homeobox gene region. HOXD-AS1 maps to chromosome 2q31.1, and it is transcribed in the antisense mode from the HOXD cluster between HOXD1 and HOXD3 [11]. The purpose of this study is to explore the function and mechanism of HOXD-AS1 in CCA progression.

As another type of ncRNAs, microRNAs (miRNAs) are also an important regulator of tumorigenesis and development [12]. At the post-transcriptional level, miRNAs can bind to the 3’UTR of various protein-coding mRNAs through complementary binding sites, thereby restraining their expression by RNA-induced silencing complex [13]. In addition, miRNAs can bind to lncRNAs through complementary binding sites. Thus, lncRNAs can modulate the expression of mRNAs by competing with them for binding intermediary miRNAs. MiR-520c-3p is mainly expressed in human tumor tissues, and it is involved in tumor pathological process mainly by the ceRNA mechanism [14]. For instance, HOXA cluster antisense RNA 2 (HOXA-AS2) competitively binds to miR-520c-3p in hepatocellular carcinoma to accelerate tumor growth [15]. MYCN proto-oncogene bHLH transcription factor (MYCN) is a member of the MYC proto-oncogene family, and this family is closely related to the regulation of embryonal development [16]. Aberrant expression of MYCN contributes to the tumorigenesis of several cancers. For example, LINC01296 increases MYCN expression by interacting with miR-5095 to promote CCA development [17]. More importantly, MYCN has been confirmed to be a drug target in MYCN-amplified neuroblastoma [18].

Cancer stem cells (CSCs) are a group of primitive tumor cells, and they are characterized by self-renewal and multilineage differentiation [19]. CSCs depend on the characteristics of stemness to induce tumorigenesis and promote tumor growth, epithelial-mesenchymal transition (EMT), chemo-resistance, and recurrence [20]. Therefore, the radical cure of tumors must completely eliminate the CSCs. SRY-box 2 (SOX2), octamer-binding protein 4 (OCT4) and Nanog are pivotal regulators in maintaining the self-renewal and pluripotency of CSCs [21, 22]. They are overexpressed in CSCs and involved in driving tumor development. In consideration of above characteristics, exploring the key molecular mechanisms in CSCs is required to thoroughly overcome cancers.

In this study, we firstly confirmed that HOXD-AS1 expression was observably elevated and linked to lymph node invasion, advanced TNM stage and poor prognosis in CCA. Specificity protein 1 (SP1)-induced HOXD-AS1 promoted tumor viability, metastasis, EMT, stemness and chemo-resistance *in vitro* and *in vivo* by sponging miR-520c-3p to increase oncogene MYCN. Taken together, HOXD-AS1 serves as a cancer-promoting gene, and SP1/HOXD-AS1/miR-520c-3p/MYCN pathway exerts vital regulatory function in initiation and progression of CCA.

## RESULTS

### HOXD-AS1 was upregulated and represented poor prognosis in CCA

**Figure 1:**
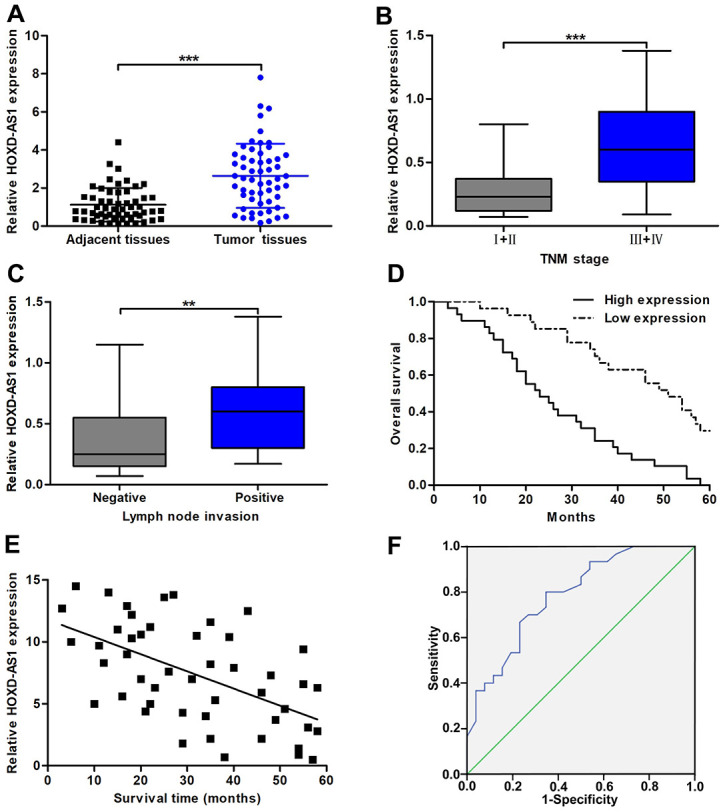
**The expression of HOXD-AS1 and its correlation with clinicopathological characteristics and prognosis.** (**A**) HOXD-AS1 expression in CCA tissues and paired adjacent nontumor bile duct tissues was detected by qRT-PCR. (**B**) HOXD-AS1 expression in tissues at TNM I+II stage and TNM III+IV stage was detected by qRT-PCR. (**C**) HOXD-AS1 expression in tissues with lymph node invasion and without lymph node invasion was detected by qRT-PCR. (**D**) CCA patients were divided into two groups according to average value of HOXD-AS1 expression. Overall survival was evaluated between high and low HOXD-AS1 expression groups by using Kaplan-Meier method and log-rank test. (**E**) The correlation between relative HOXD-AS1 expression and survival time of CCA patients was assessed by Pearson correlation analysis. (**F**) The sensitivity and specificity of HOXD-AS1 as a prognostic marker were analyzed by ROC curve. ^**^P < 0.01, ^***^P < 0.001.

HOXD-AS1 has been reported to be highly expressed in a variety of digestive system tumors, such as gastric cancer, hepatocellular carcinoma, and colorectal carcinoma. However, its function in CCA is still unclear. After database The Cancer Genome Atlas (TCGA) analysis, we found that the expression of HOXD-AS1 was upregulated in CCA tissues (*P* < 0.001, Supplementary Figure 1A). Therefore, we intend to further confirm the functional role of HOXD-AS1 in CCA through basic experiments. As analyzed in TCGA database, we confirmed that HOXD-AS1 was significantly overexpressed in CCA tissues compared with that in paired adjacent nontumor bile duct tissues (Figure 1A). The analysis of the clinicopathological correlation confirmed that increased HOXD-AS1 was dramatically associated with lymph node invasion and advanced TNM stage (Figure 1B, 1C). However, HOXD-AS1 was not associated with other clinicopathological features, including age, gender, tumor location, histological type, differentiation grade, HBV infection, serum CA19-9 level, and serum CEA level (Table 1). By dividing all patients into high HOXD-AS1 expression group and low expression group, survival correlation was also analyzed in this study. The results showed that patients in increased HOXD-AS1 group presented worse overall survival (OS) than those in decreased HOXD-AS1 group (log rank *P* < 0.001, Figure 1D). In addition, Pearson correlation analysis confirmed that HOXD-AS1 expression was negatively associated with the survival of CCA patients (*r* = -0.5592, *P* < 0.001, Figure 1E). These findings suggested that HOXD-AS1 was a regulator of the progression of CCA. Hence, we further assessed the prognostic value of HOXD-AS1 by univariate and multivariate analyses. The univariate analysis revealed that high HOXD-AS1 expression, advanced TNM stage, and lymph node invasion were associated with prognosis of CCA patients. The multivariate analysis uncovered that high HOXD-AS1 expression and advanced TNM stage were independent risk factors of prognosis of CCA patients (Table 2). As shown in Figure 1F, the receiver operating characteristic curve (ROC) analysis indicated that the value of area under curve (AUC) of HOXD-AS1 as a prognostic marker was 0.786 (95% CI: 0.668-0.904) with 80% sensitivity and 65.4% specificity (*P* < 0.001).

**Table 1:** Correlation between HOXD-AS1 expression and clinicopathological characteristics of CCA patients.

**Clinicopathological parameters**	**Total cases (*n* = 56)**	**HOXD-AS1 expression**		***P*-value**
		**Low (*n* = 27)**	**High (*n* = 29)**	
Age (years)				
< 60	19	10	9	0.635
≥ 60	37	17	20	
Gender				
Male	25	11	14	0.571
Female	31	16	15	
Tumor location				
Intrahepatic	21	12	9	0.300
Extrahepatic	35	15	20	
Histological type				
Adenocarcinoma	51	24	27	0.580
Non-adenocarcinoma	5	3	2	
Differentiation grade				
Well/moderate	26	15	11	0.186
Poor/undifferentiated	30	12	18	
TNM stage				
I-II	24	17	7	0.003**
III-IV	32	10	22	
Lymph node invasion				
Yes	36	13	23	0.015*
No	20	14	6	
HBV infection				
Positive	18	8	10	0.697
Negative	38	19	19	
Serum CA19-9 level				
> 37 U/ml	36	15	21	0.188
≤ 37 U/ml	20	12	8	
Serum CEA level				
> 5 ng/ml	33	14	19	0.299
≤ 5 ng/ml	23	13	10	

Note. ^*^P < 0.05, ^**^P < 0.01. HOXD-AS1, HOXD cluster antisense RNA 1; CCA, cholangiocarcinoma.

**Table 2:** Univariate and multivariate analyses for overall survival of CCA patients.

**Variables**	**Univariate analysis**			**Multivariate analysis**		
	**HR**	**95% CI**	***P*-value**	**HR**	**95% CI**	***P*-value**
Age (years) ≥ 60 *vs*. < 60	1.302	0.714-2.374	0.390			
Gender Male *vs*. Female	0.857	0.483-1.521	0.598			
Tumor location Extrahepatic *vs*. Intrahepatic	1.405	0.776-2.543	0.261			
Histological type Adenocarcinoma *vs*. Non-adenocarcinoma	0.750	0.269-2.091	0.582			
Differentiation grade Poor/undifferentiated *vs*. Well/moderate	1.520	0.856-2.699	0.153			
HBV infection Positive *vs*. Negative	0.822	0.446-1.515	0.530			
Serum CA19-9 level > 37 U/ml *vs*. ≤ 37 U/ml	1.625	0.895-2.949	0.111			
Serum CEA level > 5 ng/ml *vs*. ≤ 5 ng/ml	1.429	0.799-2.555	0.228			
TNM stage III-IV *vs*. I-II	2.090	1.173-3.724	0.012*	2.210	1.241-3.936	0.007**
Lymph node invasion Yes *vs*. No	1.955	1.063-3.596	0.031*	1.800	0.981-3.303	0.058
HOXD-AS1 expression Low *vs*. High	2.124	1.193-3.780	0.010*	2.411	1.350-4.307	0.003**

Note. ^*^P < 0.05, ^**^P < 0.01. CCA, cholangiocarcinoma; HR, hazard ratio; CI, confidence interval; HOXD-AS1, HOXD cluster antisense RNA 1.

### HOXD-AS1 promoted CCA cell proliferation, migration, invasion and EMT

**Figure 2:**
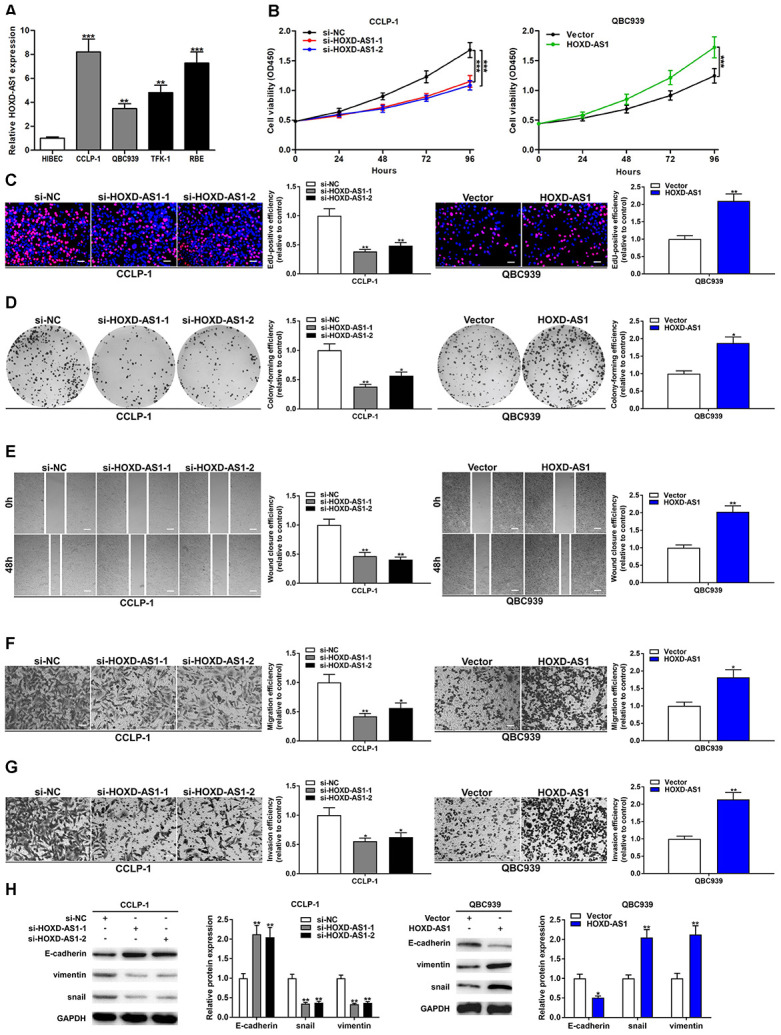
**Upregulated HOXD-AS1 facilitated the viability, migration and invasion of CCA cells.** (**A**) The expression of HOXD-AS1 in CCLP-1, QBC939, TFK-1, RBE cells and normal HIBEC. (**B**) CCK-8 proliferation curves were drawn to show the impact of HOXD-AS1 on cellular proliferation. (**C**) EdU assay was performed to detect the effect of HOXD-AS1 on cellular proliferation. (**D**) The effect of HOXD-AS1 on cell colonies was revealed by colony formation assays. (**E**) The wound closure of CCA cells was evaluated by wound healing assays. (**F**) Transwell assays displayed that migrating cells were decreased in si-HOXD-AS1 cells and increased in QBC939 cells transfected with HOXD-AS1 plasmid. (**G**) The invasive ability of CCA cells was confirmed by transwell assay. (**H**) EMT-related proteins including epithelial marker (E-cadherin) and mesenchymal markers (snail and vimentin) were measured via western blot. ^*^P < 0.05, ^**^P < 0.01, ^***^P < 0.001.

According to the high expression of HOXD-AS1 in CCA tissues, we further detected its expression in CCA cells. As shown in Figure 2A, HOXD-AS1 expression was also upregulated in CCLP-1, QBC939, TFK-1 and RBE in comparison with that in HIBEC. According to the results of quantitative real-time polymerase chain reaction (qRT-PCR), CCLP-1 and QBC939 were selected for the next cytology experiments. The knockdown efficiency and amplification efficiency of HOXD-AS1 in CCLP-1 and QBC939 were shown in Supplementary Figure 1B. Three specific siRNAs were designed to knock down HOXD-AS1, and si-HOXD-AS1-1 and si-HOXD-AS1-2 were chosen for the further experiments according to their knockdown efficiency. We tested the effect of HOXD-AS1 on the proliferation of CCA cells by loss- and gain-of-function experiments. The cell counting kit-8 (CCK-8) assays showed that HOXD-AS1 knockdown significantly suppressed proliferation of CCLP-1 cells, and upregulated HOXD-AS1 facilitated proliferation of QBC939 cells in comparison with negative controls, respectively (Figure 2B). In 5-ethynyl-2’-deoxyuridine (EdU) incorporation assays, HOXD-AS1 knockdown significantly reduced the number of red stains in CCLP-1 cells, while its overexpression increased the red stain numbers in QBC939 cells (Figure 2C). These results suggested that HOXD-AS1 promoted proliferation of CCA cells. In addition, knocking down HOXD-AS1 inhibited the colony formation ability of CCA cells, while its high expression achieved opposite result (Figure 2D).

Metastasis occurs in advanced stages of tumors, thereby increasing the difficulty of treatment. And the metastasis of CCA occurs earlier. Therefore, we detected the effect of HOXD-AS1 on the metastasis ability of CCA cells by wound healing and transwell assays. As uncovered in Figure 2E, silencing HOXD-AS1 suppressed CCLP-1 motility and increased HOXD-AS1 promoted the migration of QBC939 cells. Transwell assay without Matrigel also corroborated that HOXD-AS1 facilitated CCA cell migration (Figure 2F). Transwell assay with Matrigel confirmed that HOXD-AS1 knockdown reduced the number of invasive cells, and high HOXD-AS1 expression promoted invasion of CCA cells (Figure 2G). EMT is an imperative process of tumor metastasis. In this study, we confirmed that knocking down HOXD-AS1 increased epithelial marker E-cadherin expression and reduced the expression of mesenchymal markers (snail and vimentin) in CCLP-1 cells. Whereas its overexpression restrained E-cadherin expression and promoted the expression of snail and vimentin in QBC939 cells (Figure 2H).

### HOXD-AS1 contributed to stemness maintenance and chemo-resistance in CCA

**Figure 3:**
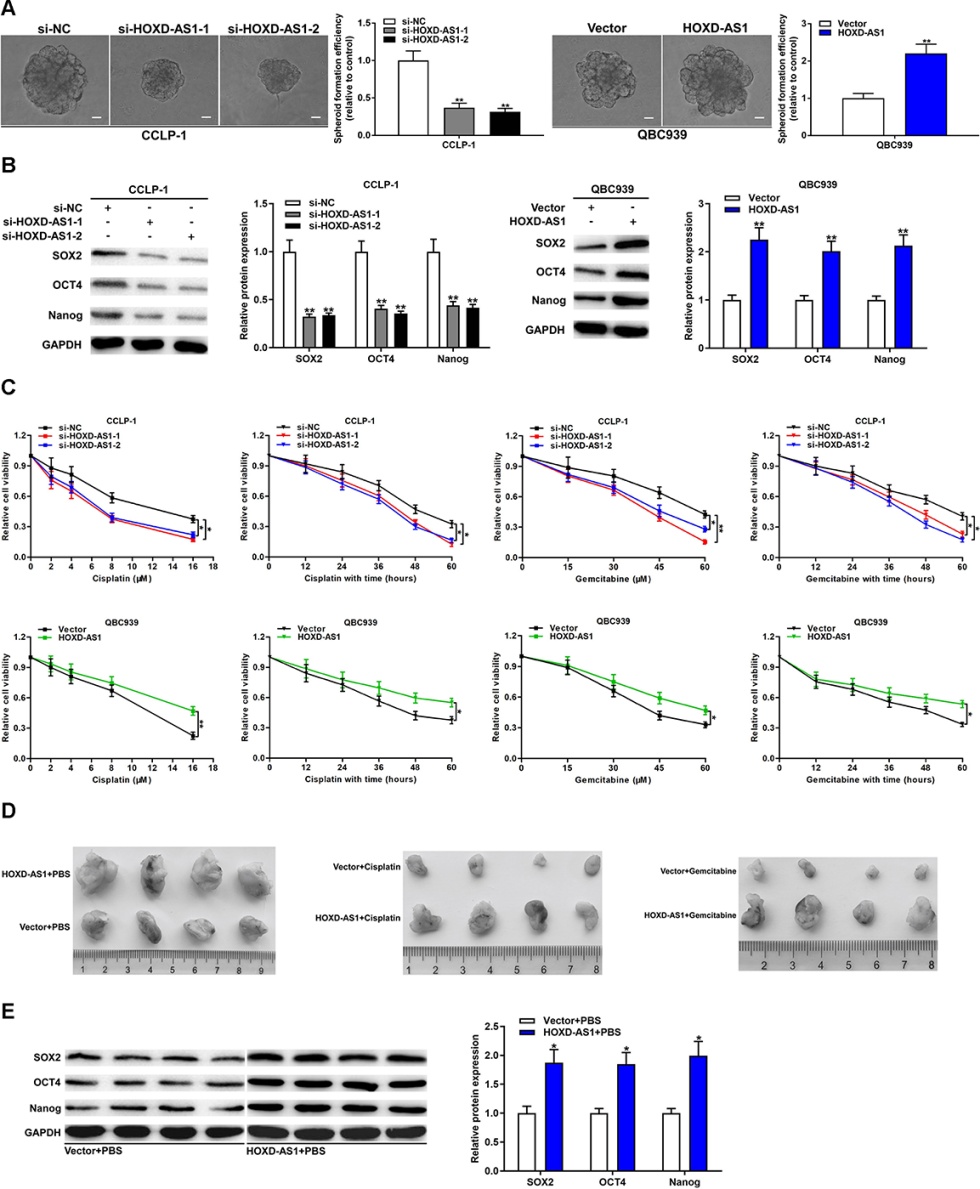
**HOXD-AS1 promoted stemness maintenance and chemo-resistance in CCA.** (**A**) Spheroid forming abilities of transfected QBC939 and CCLP-1 cells were evaluated by spheroid formation assay. (**B**) The effect of HOXD-AS1 on stem markers (SOX2, OCT4, Nanog) was detected by western blot. (**C**) The effect of HOXD-AS1 on the treatment of cisplatin and gemcitabine was measured by CCK-8 assay. (**D**) Mice were subcutaneously injected with QBC939 cells transfected with HOXD-AS1 plasmid and intraperitoneally injected with PBS, cisplatin or gemcitabine. (**E**) The stem markers (SOX2, OCT4, Nanog) in xenograft tumors were measured by western blot. ^*^P < 0.05, ^**^P < 0.01.

We measured the correlation between HOXD-AS1 and tumor stemness as well as its effect on chemo-resistance of CCA. CSCs possess the ability to form a tumor spheroid. As displayed in Figure 3A, spheroid formation assays certified that HOXD-AS1 knockdown inhibited the formation of tumor spheroid in CCLP-1 cells, whereas HOXD-AS1 overexpression promoted the spheroid formation of CCA cells. Furthermore, we examined the effect of HOXD-AS1 on the expression of stem markers in CCA cells. The results showed that silencing HOXD-AS1 inhibited the expression of SOX2, OCT4 and Nanog in CCLP-1 cells, while its upregulation promoted their expression in QBC939 cells (Figure 3B). For chemo-resistance detection, transfected CCA cells were treated with cisplatin or gemcitabine in dose-dependent and time-dependent manners. As shown in Figure 3C, CCK-8 assays revealed that knocking down HOXD-AS1 enhanced the sensitivity of CCLP-1 cells to cisplatin and gemcitabine, whereas elevated HOXD-AS1 reinforced resistance effect of QBC939 cells on treatment of cisplatin and gemcitabine.

We further explored the effect of HOXD-AS1 on chemo-resistance *in vivo* by applying nude mice model. QBC939 cells transfected with HOXD-AS1 plasmid or vector were subcutaneously injected into mice. Meanwhile, the mice were treated with phosphate-buffered saline (PBS), cisplatin or gemcitabine by intraperitoneal injection. As shown in Figure 3D, HOXD-AS1 overexpression significantly promoted tumor growth compared with the vector group, and treatment with cisplatin or gemcitabine markedly restrained tumor growth compared with PBS controls. More importantly, HOXD-AS1 heightened the chemo-resistance of tumors on treatment of cisplatin and gemcitabine *in vivo*. In addition, we detected the expression of stem markers in HOXD-AS1 + PBS and vector + PBS groups. The results displayed that HOXD-AS1 increased the expression of SOX2, OCT4 and Nanog *in vivo* compared with controls (Figure 3E).

### Transcription factor SP1 directly promoted HOXD-AS1 transcription

**Figure 4:**
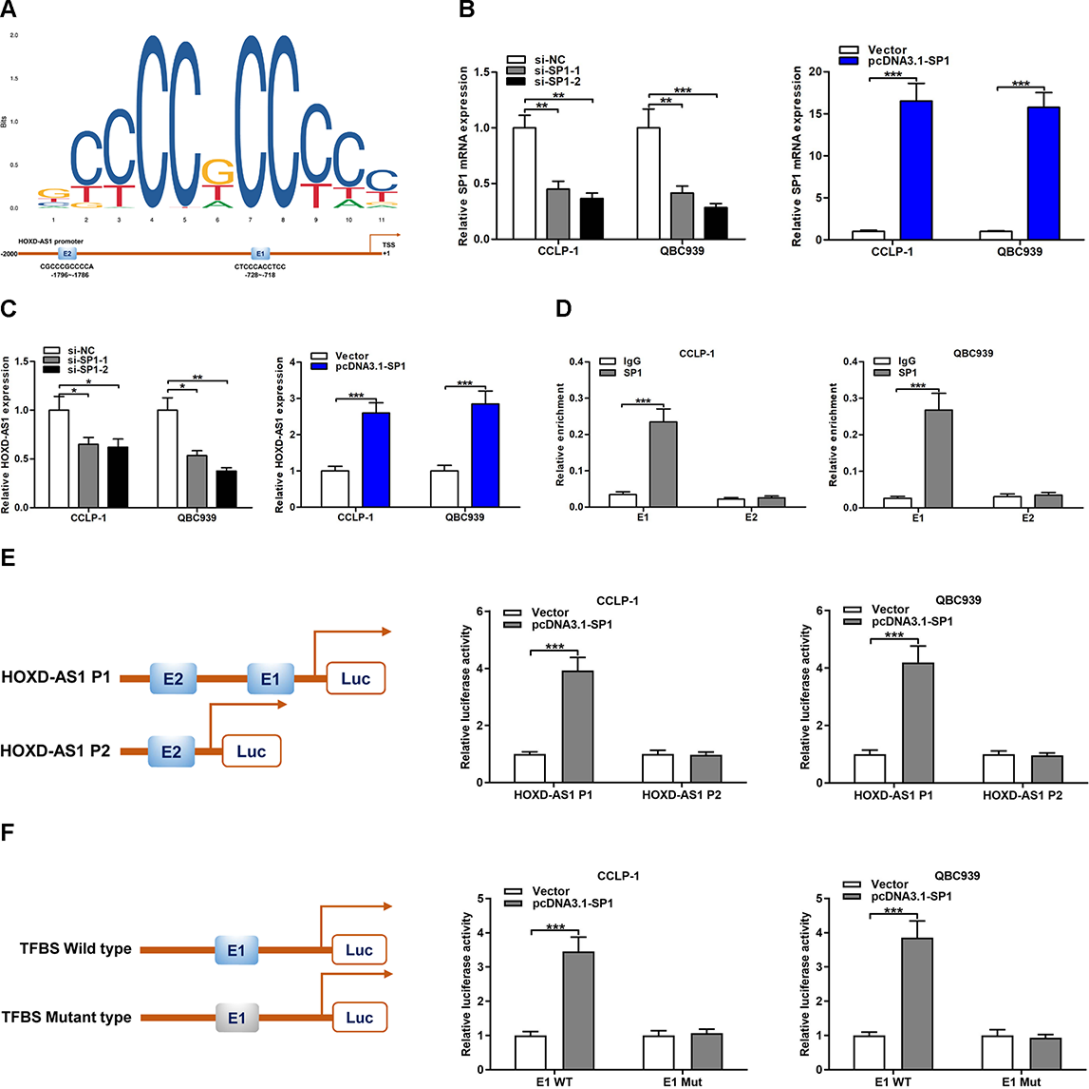
**HOXD-AS1 was induced by transcription factor SP1.** (**A**) SP1 sequence and binding sites (E1 and E2) to HOXD-AS1 promoter region were predicted by using JASPAR database (http://jaspar.genereg.net/). (**B**) The knockdown efficiency and amplification efficiency of SP1 in CCLP-1 and QBC939 were detected by qRT-PCR. (**C**) Upregulated SP1 facilitated HOXD-AS1 expression and decreased SP1 restrained HOXD-AS1 expression in QBC939 and CCLP-1 cells. (**D**) ChIP assays were performed to confirm the direct binding of SP1 to HOXD-AS1 promoter in QBC939 and CCLP-1 cells. (**E**) Luciferase reporter assay showed that SP1 bound to E1 rather than E2. (**F**) Luciferase reporter assay displayed that SP1 bound to E1 wild type rather than mutant type. ^*^P < 0.05, ^**^P < 0.01, ^***^P < 0.001.

SP1 was demonstrated to promote various target gene transcription by acting as a transcription factor [23]. By the JASPAR database (http://jaspar.genereg.net/) detection, two binding sites of SP1 (E1 and E2) were identified in HOXD-AS1 promoter region (Figure 4A). To further explore the regulatory function of SP1 on HOXD-AS1, SP1 was severally knocked down or amplified in CCA cells (Figure 4B). The qRT-PCR assays verified that SP1 knockdown decreased HOXD-AS1 transcription and SP1 overexpression increased HOXD-AS1 transcription in CCLP-1 and QBC939 cells (Figure 4C). To further determine the binding of SP1 to HOXD-AS1 promoter, chromatin immunoprecipitation (ChIP) assays revealed that SP1 antibody dramatically recruited the fragments of transcription factor binding site (TFBS) E1 (Figure 4D). Furthermore, luciferase reporter plasmid with E1 and E2 was activated by SP1, but luciferase reporter plasmid with E2 was not affected (Figure 4E). In addition, SP1 activated luciferase reporter plasmid with wild type E1, but luciferase reporter plasmid with mutant type E1 was not activated (Figure 4F). These results suggested that SP1 promoted HOXD-AS1 transcription by directly binding to TFBS E1 in HOXD-AS1 promoter region.

### HOXD-AS1 served as a sponge of miR-520c-3p in CCA

**Figure 5:**
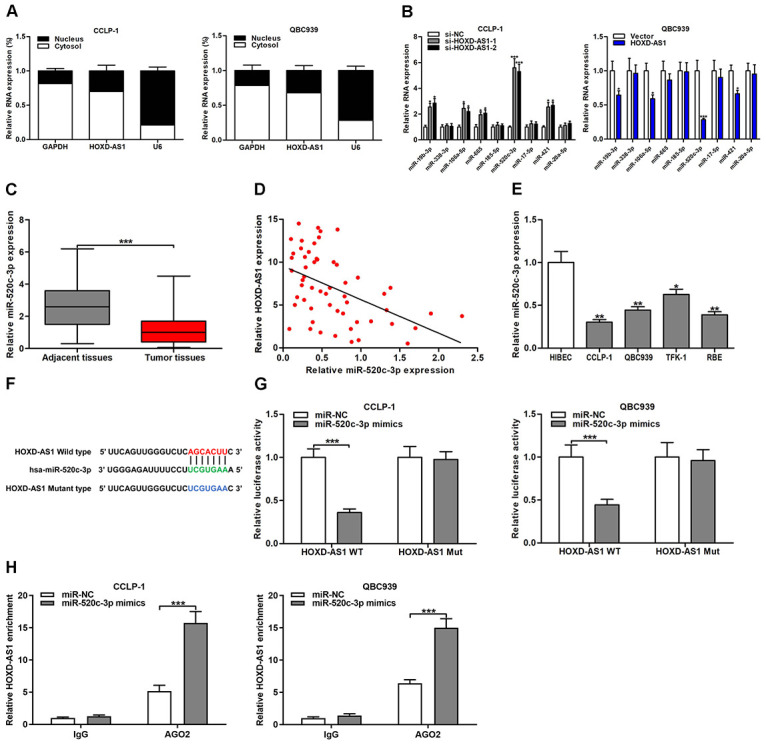
**HOXD-AS1 functioned as a sponge for miR-520c-3p in CCA cells.** (**A**) Subcellular localization of HOXD-AS1 was tested by subcellular fractionation assays. GAPDH and U6 were used as endogenous controls for cytoplasm and nucleus, respectively. (**B**) The expression levels of predicted miRNAs were detected after knocking down or amplifying HOXD-AS1 in CCLP-1 and QBC939 cells, respectively. (**C**) The expression of miR-520c-3p in CCA tissues and paired adjacent nontumor bile duct tissues. (**D**) The correlation between relative HOXD-AS1 expression and relative miR-520c-3p expression in CCA tissues. (**E**) The miR-520c-3p expression in CCA cells (CCLP-1, QBC939, TFK-1, RBE) and normal HIBEC. (**F**) Luciferase reporter plasmids were constructed with miR-520c-3p-binding site region of HOXD-AS1 sequence, including wild type and mutant type. (**G**) Luciferase reporter assays showed that cotransfected miR-520c-3p mimics significantly inhibited luciferase activity of HOXD-AS1 wild type. (**H**) AGO2 RIP assays further suggested the binding of miR-520c-3p to HOXD-AS1. ^*^P < 0.05, ^**^P < 0.01, ^***^P < 0.001.

To explore the downstream regulation mechanism of HOXD-AS1, we first detected its localization in CCA cells. Subcellular fractionation assays attested that HOXD-AS1 was mainly expressed in cytoplasm compared with nucleus of CCA cells (Figure 5A). Hence, HOXD-AS1 may play a regulatory role at the post-transcriptional level. The ceRNA mechanism is the main regulatory manner of lncRNAs at post-transcriptional level. Therefore, we predicted the downstream target of HOXD-AS1 on the basis of ceRNA mode through online database StarBase v3.0 (http://starbase.sysu.edu.cn/). As shown in Figure 5B, HOXD-AS1 interacted with multiple miRNAs by acting as a molecular sponge, and miR-520c-3p was selected for further mechanism research according to the results of qRT-PCR. The knockdown efficiency of inh-miR-520c-3p and amplification efficiency of miR-520c-3p mimics in CCLP-1 and QBC939 were shown in Supplementary Figure 1C.

MiR-520c-3p expression was significantly downregulated in CCA tissues compared with that in paired adjacent nontumor bile duct tissues (Figure 5C). Downregulated miR-520c-3p was negatively correlated with HOXD-AS1 expression in CCA tissues (*r* = -0.4846, *P* < 0.001, Figure 5D). Moreover, miR-520c-3p was markedly downexpressed in CCA cells as well (Figure 5E). The binding site of miR-520c-3p and HOXD-AS1 was detected by StarBase v3.0 (Figure 5F). The HOXD-AS1 wild-type and mutant-type binding sites were designed and cloned into the luciferase reporter plasmids. The cotransfected miR-520c-3p mimics significantly inhibited luciferase activity of the wild-type plasmid group, but the luciferase activity of mutant-type plasmid group was not affected. The results confirmed that HOXD-AS1 directly bound to miR-520c-3p through this binding site (Figure 5G). RNA immunoprecipitation (RIP) assays showed that HOXD-AS1 and miR-520c-3p enrichments were higher in AGO2 groups with miR-520c-3p mimics than controls (Figure 5H, Supplementary Figure 1D), further suggesting the targeted binding of HOXD-AS1 to miR-520c-3p.

### MYCN was a direct target of miR-520c-3p in CCA

**Figure 6:**
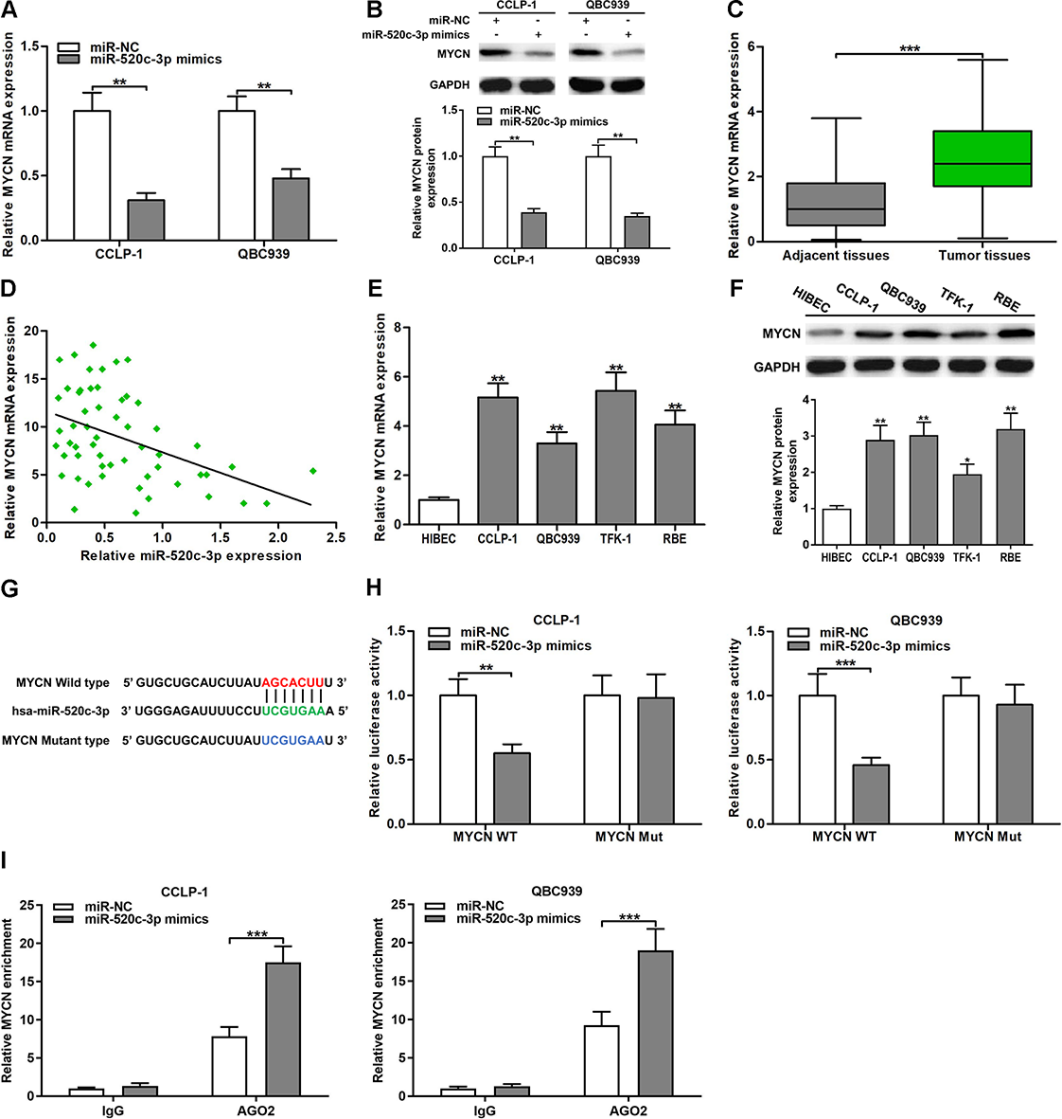
**MiR-520c-3p was a direct regulator of MYCN in CCA cells.** (**A**) MiR-520c-3p restrained MYCN mRNA expression certified by qRT-PCR. (**B**) MiR-520c-3p refrained MYCN protein expression testified via western blot. (**C**) The expression of MYCN mRNA in CCA tissues and paired adjacent nontumor bile duct tissues. (**D**) The correlation between relative MYCN mRNA expression and relative miR-520c-3p expression in CCA tissues. (**E**) The MYCN mRNA expression in CCLP-1, QBC939, TFK-1, RBE and normal HIBEC. (**F**) The MYCN protein expression in CCA cells (CCLP-1, QBC939, TFK-1, RBE) and normal HIBEC. (**G**) Luciferase reporter plasmids were constructed with miR-520c-3p-binding site region of MYCN sequence, including wild type and mutant type. (**H**) The luciferase activity of MYCN wild type was observably inhibited by miR-520c-3p mimics cotransfection. (**I**) AGO2 RIP assays were conducted to further demonstrate the binding of miR-520c-3p to 3’UTR of MYCN. ^*^P < 0.05, ^**^P < 0.01, ^***^P < 0.001.

Oncogene MYCN has been testified to play a critical regulatory role in many digestive system tumors [24]. TCGA database confirmed that the expression of MYCN was increased in CCA tissues (*P* < 0.001; Supplementary Figure 1E). By online database StarBase prediction, MYCN and HOXD-AS1 had the same binding site to interact with miR-520c-3p. Therefore, we decided to verify the correlation of MYCN and miR-520c-3p. The knockdown efficiency of si-MYCN in CCLP-1 and QBC939 were shown in Supplementary Figure 1F. As displayed in Figure 6A, upregulated miR-520c-3p inhibited the expression of MYCN mRNA in CCLP-1 and QBC939 cells. Similarly, increased miR-520c-3p also suppressed MYCN protein expression (Figure 6B). By qRT-PCR analysis, MYCN expression was remarkably upregulated in CCA tissues compared with that in paired adjacent nontumor bile duct tissues (Figure 6C). Moreover, MYCN expression was negatively associated with miR-520c-3p expression (*r* = -0.4449, *P* < 0.001, Figure 6D). In addition, MYCN was also overexpressed in CCA cells both at mRNA and protein levels (Figure 6E, 6F). Subsequently, luciferase reporter assays confirmed that luciferase activity of MYCN wild type was restrained by miR-520c-3p mimics, but miR-520c-3p mimics didn’t affect luciferase activity of MYCN mutant type (Figure 6G, 6H). Besides, AGO2 RIP assays demonstrated that miR-520c-3p mimics dramatically enriched MYCN mRNA in CCA cells (Figure 6I). The findings indicated that miR-520c-3p directly bound MYCN 3’UTR to repress MYCN expression in CCA cells.

### SP1-induced HOXD-AS1 promoted CCA development by sponging miR-520c-3p to upregulate MYCN

**Figure 7:**
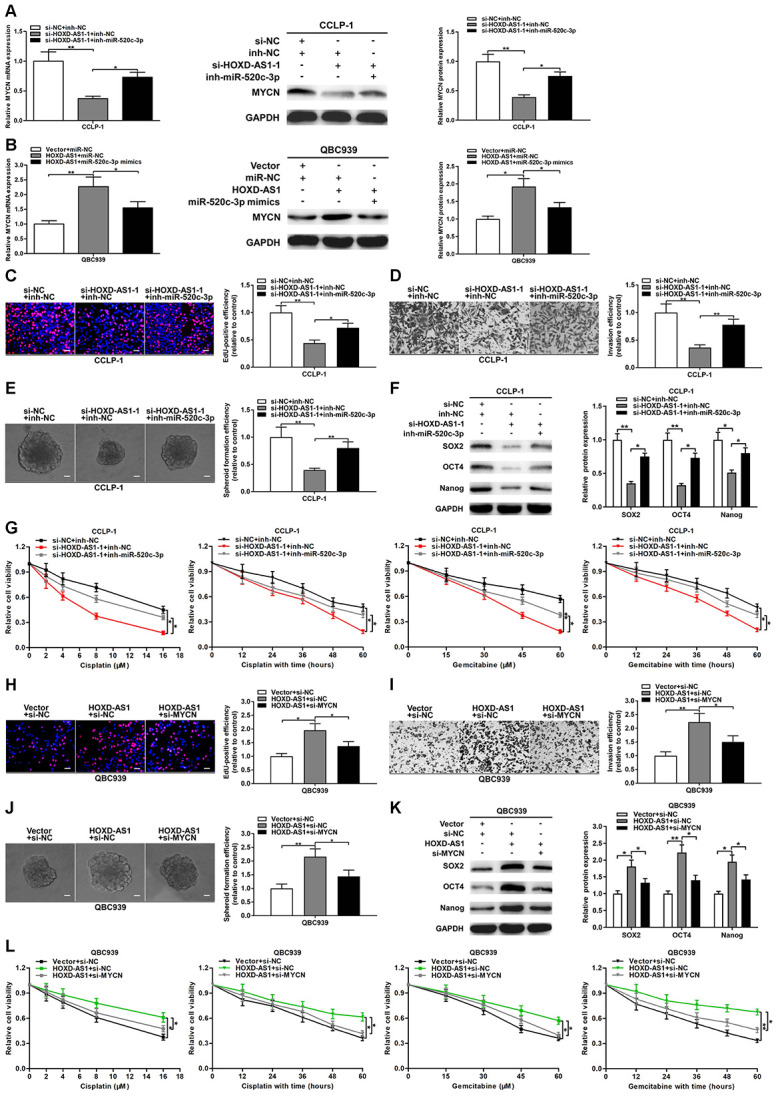
**HOXD-AS1 boosted malignant progression of CCA through mediating miR-520c-3p/MYCN.** (**A**) MYCN downexpression caused by HOXD-AS1 knockdown was saved by silencing miR-520c-3p. (**B**) MYCN overexpression caused by HOXD-AS1 amplification was saved by increasing miR-520c-3p. (**C**–**G**) Rescue assays of EdU, transwell, spheroid formation, stem marker analysis and chemo-resistance certified that inhibition of proliferation, invasion, stemness maintenance and chemo-resistance induced by knocking down HOXD-AS1 was retrieved through silencing miR-520c-3p in CCLP-1 cells, respectively. (**H**–**L**) Restoration of MYCN expression rescued the promotion of proliferation, invasion, stemness maintenance and chemo-resistance generated through HOXD-AS1 amplification in EdU, transwell, spheroid formation, stem marker analysis and chemo-resistance assays, respectively. ^*^P < 0.05, ^**^P < 0.01.

To further confirm the regulation of HOXD-AS1/miR-520c-3p/MYCN in CCA, we conducted function-related rescue experiments. As shown in Figure 7A, HOXD-AS1 knockdown inhibited the expression of MYCN both at mRNA and protein levels, while simultaneously silencing miR-520c-3p could partially reverse the suppressive function of HOXD-AS1 knockdown. Likewise, HOXD-AS1 overexpression promoted MYCN expression, while upregulated miR-520c-3p could partially save the function of HOXD-AS1 overexpression (Figure 7B). EdU assay confirmed that knocking down HOXD-AS1 inhibited the proliferation of CCLP-1 cells, while miR-520c-3p knockdown could partially reverse the function of HOXD-AS1 knockdown (Figure 7C). In transwell assay, miR-520c-3p knockdown could also partially rescue the invasive inhibition induced by HOXD-AS1 knockdown (Figure 7D). In terms of stemness maintenance and chemo-resistance, silencing HOXD-AS1 restrained spheroid formation and stem marker expression, and sensitized CCLP-1 cells to treatment of cisplatin and gemcitabine, while knocking down miR-520c-3p could partially save the effect of HOXD-AS1 knockdown (Figure 7E–7G).

Whether the cancer-promoting effect mediated by HOXD-AS1 can be reversed by MYCN, we also perform rescue experiments. As displayed in Figure 7H–7I, knocking down MYCN could partially save the proliferative and invasive promotion induced by HOXD-AS1 overexpression in QBC939 cells. Besides, recovery of MYCN reversed the facilitation of stemness maintenance and chemo-resistance generated by HOXD-AS1 overexpression (Figure 7J–7L). We also detected the functional relevance of miR-520c-3p and MYCN through rescue experiments. As expected, cancer-promoting function on proliferation, invasion, stemness maintenance and chemo-resistance generated by miR-520c-3p knockdown was retrieved in part through silencing MYCN (Supplementary Figure 2). These results suggested that SP1-induced HOXD-AS1 increased oncogene MYCN by competitively binding to miR-520c-3p, thereby accelerating the malignant progression of CCA.

### HOXD-AS1/miR-520c-3p/MYCN promoted CCA tumorigenesis *in vivo*

**Figure 8:**
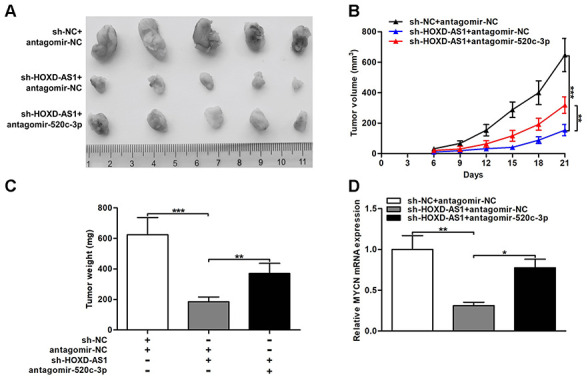
**HOXD-AS1/miR-520c-3p/MYCN contributed to CCA tumorigenesis *in vivo*.** (**A**) CCLP-1 cells cotransfected with sh-HOXD-AS1 and antagomir-520c-3p were subcutaneously injected into the posterior flanks of mice, and xenograft tumors were dissected on the 21^st^ day after injection. (**B**) Tumor volumes were calculated every 3 days throughout the course of tumor growth. (**C**) Tumor weights were measured after excision. (**D**) MYCN mRNA expression in xenograft tumors of the three groups (sh-NC/antagomir-NC, sh-HOXD-AS1/antagomir-NC, sh-HOXD-AS1/antagomir-520c-3p). ^*^P < 0.05, ^**^P < 0.01, ^***^P < 0.001.

The effect of HOXD-AS1 on CCA tumorigenesis *in vivo* was evaluated through the nude mice model. As displayed in Figure 8A–8C, HOXD-AS1 knockdown repressed the tumor volumes and the tumor weights, while cotransfected antagomir-520c-3p could partially reverse the repressive effect of HOXD-AS1 knockdown in nude mice. In addition, the expression of MYCN in tumor xenograft was detected by qRT-PCR. The results showed that knocking down HOXD-AS1 restrained MYCN expression in tumor xenograft, but silencing miR-520c-3p could partially rescue the suppressive function caused by HOXD-AS1 knockdown (Figure 8D). These findings indicated that HOXD-AS1/miR-520c-3p/MYCN contributed to CCA tumorigenesis *in vivo*.

## DISCUSSION

The prognosis of CCA is quite poor due to lack sensitive diagnostic markers and efficient therapy methods. Investigating key molecular abnormalities in the pathological process of CCA is urgently needed to improve treatment of CCA. The discovery of lncRNAs provides a valuable study direction for tumorigenesis and development at the molecular level. Accumulating evidence confirmed that lncRNAs exhibited a crucial function in the occurrence and development of CCA [25]. For example, lncRNA-ZFAS1 facilitated proliferation and invasion of CCA cells by regulating miR-296-5p/USF1 [26]; NNT-AS1 served as a ceRNA of miR-485 to promote CCA progression [27]. Among all tumor-related lncRNAs, HOXD-AS1 exhibited excellent characteristics to be a carcinogen. For instance, HOXD-AS1 promoted hepatocellular carcinoma metastasis by ceRNA manner [28]; HOXD-AS1 was related to poor clinicopathological parameters and represented an independent prognostic factor for patients with colorectal cancer [9]. In this study, we explored the regulatory role of HOXD-AS1 in CCA for the first time.

The TCGA database showed that the expression of HOXD-AS1 was increased in CCA tissues. By qRT-PCR analysis, HOXD-AS1 expression was upregulated in 56 pairs of CCA tissues, and its upregulation was significantly related to lymph node invasion and advanced TNM stage. Moreover, survival analysis showed that CCA patients with high expression of HOXD-AS1 had a worse clinical outcome, and HOXD-AS1 overexpression was negatively correlated with survival time. These results suggested that HOXD-AS1 played an important cancer-promoting role in the occurrence and development of CCA. To further analyze the prognostic value of HOXD-AS1 in CCA, multivariate analysis confirmed that HOXD-AS1 was an independent risk factor for the prognosis of CCA patients. And HOXD-AS1 as a prognostic biomarker possessed high sensitivity and specificity. These findings indicated that HOXD-AS1 had significant value in evaluation of diagnosis and prognosis in CCA.

Proliferation and metastasis are the hallmark of the malignant biological behavior of tumors. The present study demonstrated that HOXD-AS1 promoted the proliferation, metastasis and EMT process of CCA cells by gain- and loss-of-function experiments. Moreover, HOXD-AS1 also promoted the growth of subcutaneous xenograft tumors in nude mice. These results suggested that HOXD-AS1, as an oncogene, facilitated the proliferation and metastasis of CCA both *in vitro* and *in vivo*. CSCs contribute to tumor metastasis, recurrence and drug resistance. To completely cure the tumors, CSCs must be thoroughly eliminated. In this study, HOXD-AS1 promoted the stemness maintenance of CCA confirmed by tumor spheroid formation assay and stem marker detection. Furthermore, we detected the effect of HOXD-AS1 on chemo-resistance of CCA. Both *in vitro* and *in vivo*, HOXD-AS1 promoted the resistance of chemotherapy of cisplatin and gemcitabine in CCA. These findings suggested that HOXD-AS1 promoted malignant biological behavior of CCA, and it had great potential in the treatment of CCA.

Oncogene MYCN has been reported to perform a critical regulatory role in a variety of tumors. Its function in tumor stemness maintenance is also widely investigated. In neuroblastoma, MYCN interacted with OCT4 to promote the function of CSCs [29]. Study also confirmed that MYCN and SOX2 were involved in the mechanism of neuroblastoma formation [30]. By JASPAR analysis, SP1 could bind to the promoter of HOXD-AS1. By StarBase prediction, we found that HOXD-AS1 could compete with MYCN to bind miR-520c-3p. Moreover, TCGA confirmed the upregulation of MYCN in CCA. By qRT-PCR detection, we also verified the overexpression of MYCN and the downexpression of miR-520c-3p in CCA tissues. Furthermore, we confirmed that SP1-mediated HOXD-AS1 as a ceRNA upregulated MYCN by competitively binding to miR-520c-3p. Rescue experiments further confirmed that HOXD-AS1 promoted the progression of CCA through SP1/HOXD-AS1/miR-520c-3p/MYCN pathway.

In conclusion, the present study confirmed that HOXD-AS1 was overexpressed and correlated with poor clinicopathological parameters and prognosis in CCA. In addition, HOXD-AS1 was promoted by transcription factor SP1. SP1-induced HOXD-AS1 promoted CCA proliferation, migration, invasion, EMT, stemness maintenance and chemo-resistance by the mechanism of regulating miR-520c-3p/MYCN. Taken together, HOXD-AS1 has a favorable prospect to be a tumor biomarker or therapeutic target in CCA.

## MATERIALS AND METHODS

### Tissue samples and clinical data

CCA tissues and paired adjacent nontumor bile duct tissues were collected from 56 patients who underwent surgical operation at The 2^nd^ Affiliated Hospital of Harbin Medical University. The tissue specimens were immediately frozen after resection and then stored in liquid nitrogen. Two professional pathologists were requested to identify all specimens. Patients who used radiotherapy and chemotherapy before surgery had been excluded in this study. This investigation was approved by the Ethics Committee of The 2^nd^ Affiliated Hospital of Harbin Medical University. All the patients in this study signed written informed consent.

### Cell culture and transfection

Dulbecco’s modified Eagle’s medium (DMEM) and Roswell Park Memorial Institute-1640 (RPMI-1640) supplemented with 10% fetal bovine saline (FBS; Invitrogen, Carlsbad, CA) were utilized to culture the cells in a humidified condition with 5% CO_2_ and 37^°^C. One normal human biliary epithelial cell HIBEC and four human CCA cell lines (CCLP-1, QBC939, TFK-1, RBE) were employed in this study. RBE was purchased from the Cell Bank of Chinese Academy of Sciences (Shanghai, China), and other cell lines were stored in our laboratory. All cells were passed for less than six months. The siRNA/si-NC, shRNA/sh-NC, antagomir/antagomir-NC, inhibitor/inh-NC, mimics/miR-NC and pcDNA3.1/pcDNA3.1-NC (GenePharma, Shanghai, China) were designed and purchased for knockdown and overexpression by applying Lipofectamine 3000 (Invitrogen) in the light of the manufacturer’s directions. All the sequences for transfection are presented in Supplementary Table 1.

### qRT-PCR analysis

Total RNA was extracted by using TRIzol reagent (Invitrogen). Transcriptor First Strand cDNA Synthesis Kit (Roche, Penzberg, Germany) was applied for reverse transcription of total RNA. The qRT-PCR of 20 μl volume was implemented through using FastStart Universal SYBR Green Master (Roche) and C1000 Thermal Cycler (Bio-Rad, Hercules, CA). The 2^-ΔΔCt^ method was utilized to calculate the relative expression level. U6 and glyceraldehyde 3-phosphate dehydrogenase (GAPDH) were used to be internal controls for normalization. All the primer sequences are listed in Supplementary Table 1.

### Western blot

Radio immunoprecipitation assay (RIPA) lysis buffer (Beyotime, Beijing, China) was utilized to break corresponding samples. Bicinchoninic acid (BCA) protein assay kit (Beyotime) was applied to mensurate the protein concentration. 12% sodium dodecyl sulfate-polyacrylamide gel electrophoresis (SDS-PAGE) and 0.45 μm polyvinylidene fluoride (PVDF) membrane (Millipore, Billerica, MA) were used to separate and transferc target protein, respectively. Following blocking, diluent primary and secondary antibodies (Cell Signaling Technology, Danvers, MA) were utilized to incubate the membranes sequentially. BeyoECL kit (Beyotime) was employed to visualize the protein bands. GAPDH was applied as an endogenous control. The following primary antibodies were utilized: anti-E-cadherin (Cell Signaling Technology), anti-vimentin (Cell Signaling Technology), anti-snail (Cell Signaling Technology), anti-SOX2 (Abcam, Cambridge, MA), anti-OCT4 (Abcam), anti-Nanog (Abcam), anti-GAPDH (Abcam), and anti-MYCN (Abcam).

### Cell viability assays

Transfected cells were seeded into 96-well plates in 100 μl complete culture medium. The samples were incubated with 10 μl/well CCK-8 reagent (Dojindo, Kumamoto, Japan) for 2 h prior to each test. A microplate reader (Tecan, Männedorf, Switzerland) was employed to measure 450 nm absorbance every 24 h until 96 h.

Transfected cells were cultivated with 100 μl EdU diluent (Ribobio, Guangzhou, China) for 2 h. The cell samples were fixed in paraformaldehyde and sequentially stained with Apollo 567 working solution as well as Hoechst 33342 reaction solution in the dark. A fluorescence microscope (Leica, Wetzlar, Germany) was utilized to catch images.

Colony formation assay was employed to examine colony-forming ability of CCA cells. Transfected cells were equably seeded into 6-well plates and maintained in an incubator for 12 days. The cellular colonies were fixed in paraformaldehyde and stained with crystal violet (Beyotime). The colony numbers were reckoned via visual observation.

### Migration and invasion assays

Wound healing assay was performed to estimate motility of CCA cells. Transfected cells with 80%-90% confluence were linearly scratched on the surface and cultured with serum-free medium. Cellular migration was assessed at 0 h and 48 h by gauging wound distance.

Transwell chambers (Corning, Corning, NY) precoated without and with Matrigel (BD, San Jose, CA) were used to test the capability of migration and invasion, respectively. Treated cells were evenly planted into the upper chamber with serum-free medium, and complete culture medium was added into the lower chamber. After cultivation for 24 h, the upper cells were wholly erased and the remaining cells were stained with crystal violet. An inverted optical microscope (Leica) was used to observe and calculate the colored cells.

### Spheroid formation assay

24-well ultra-low attachment plates (Corning) were utilized to cultivate CCA cells in serum-free DMEM/F12 medium supplemented with 1× B27, 20 ng/ml epidermal growth factor (EGF), 20 ng/ml basic fibroblast growth factor (bFGF, Invitrogen), 100 U/ml penicillin and 0.1 mg/ml streptomycin (Beyotime). Spheroid number and size were evaluated after 10 days under an inverted optical microscope (Leica).

### Chemo-resistance assay

Transfected cells were treated by using cisplatin (Sigma, St. Louis, MO) or gemcitabine (MedChem Express, Monmouth Junction, NJ) with concentration gradient or time gradient. CCK-8 assay was performed to measure the cytotoxicity of cisplatin and gemcitabine. In time gradient assays, the concentration of cisplatin and gemcitabine was 4 μM and 20 μM, respectively.

For *in vivo* experiment, 5-week-old female BALB/c nude mice were subcutaneously injected with transfected QBC939 in posterior flanks. After one week, PBS, cisplatin (5 mg/kg) or gemcitabine (50 mg/kg) were applied to intraperitoneally inject into mice twice a week for 2 weeks. Tumor volumes were calculated every 3 days by using the formula 0.5 × length × width^2^. All mice were euthanized after 3 weeks.

### ChIP assay

The binding of transcription factor to promoter was corroborated by ChIP assay (Millipore) according to producer’s recommendations. Cells were fixed through using formaldehyde to produce cross-linked protein and DNA, and then chromatin fragments were achieved by using sonication. Specific antibody was utilized to generate immunoprecipitations and IgG (Millipore) was regarded as negative control. The recuperated DNA fragments were evaluated via qRT-PCR. Supplementary Table 1 contained all primer sequences.

### Luciferase reporter assay

Luciferase reporter plasmids (Promega, Madison, WI) were constructed with wild type and mutant type, respectively. Firefly luciferase represented the primary reporter that monitored the binding of protein/miRNA to cloned target sequences. Renilla luciferase was regarded as a control reporter for normalization. The luciferase reporter plasmids and regulating factors were cotransfected into CCA cells through applying Lipofectamine 3000 reagent. After 48 h, luciferase activity was measured via a dual luciferase reporter assay kit (Promega).

### Subcellular fractionation assay

A PARIS kit (Life Technologies, Carlsbad, CA) was employed to isolate cytoplasmic and nuclear components. CCA cells were lysed with cell fractionation buffer and then centrifugated to acquire upper cytoplasmic contents. The remaining deposition was disposed by applying cell disruption buffer for obtaining nuclear ingredients. The qRT-PCR was utilized to test extracted RNAs from cytoplasm and nucleus, respectively. GAPDH and U6 were separately used as cytoplasmic control and nuclear control.

### RIP assay

CCA cells were lysed by using RIP lysis buffer (Millipore). The magnetic beads linked with anti-AGO2 antibody or control IgG (Millipore) were separately applied to generate immunoprecipitations. Proteinase K was utilized to treat the immunoprecipitations, thereby acquiring purified RNA samples. The qRT-PCR was further used to analyze the extracted RNAs.

### Tumor xenograft assay

All the animal works were performed in the Animal Experimental Center of The 2^nd^ Affiliated Hospital of Harbin Medical University. All animal experiments were approved and supervised by the Animal Care and Use Committee of The 2^nd^ Affiliated Hospital of Harbin Medical University. Five-week-old female BALB/c nude mice were acquired from Vital River Laboratory Animal Technology Co., Ltd. (Beijing, China) and fed in specific-pathogen-free environment. The posterior flanks of mice were subcutaneously injected with transfected CCLP-1 cell suspension. The formula 0.5 × length × width^2^ was employed to calculate the tumor volumes every 3 days. After 21 days, all mice were euthanized and tumor weights were measured.

### Statistical analysis

GraphPad Prism 6.0 software (GraphPad Software, La Jolla, CA) and SPSS 20.0 software (IBM SPSS, Armonk, NY) were applied. Data were presented as mean ± standard deviation (SD) based on at least three independent experiments. Comparisons between groups were carried out by using Student’s *t*-test, analysis of variance (ANOVA) and Chi-square test. Kaplan-Meier method and log-rank test were employed for survival analysis. Pearson correlation was utilized for correlation analysis. Prognostic risk factors were assessed via univariate and multivariate Cox proportional hazards regression model and ROC analysis. *P* value < 0.05 was considered to be a statistically significant difference.

## Supplementary Material

Supplementary Figures

Supplementary Table 1
