# Supplementary material for: SP1-induced HOXD-AS1 promotes malignant progression of cholangiocarcinoma by regulating miR-520c-3p/MYCN
Source: Aging (Albany NY). 2020 Aug 28;12(16):16304–25. doi: 10.18632/aging.103660 (PMC7485728; doi:10.18632/aging.103660)
Supplement: Supplementary Figures [file aging-12-103660-s002..pdf]

SUPPLEMENTARY FIGURES

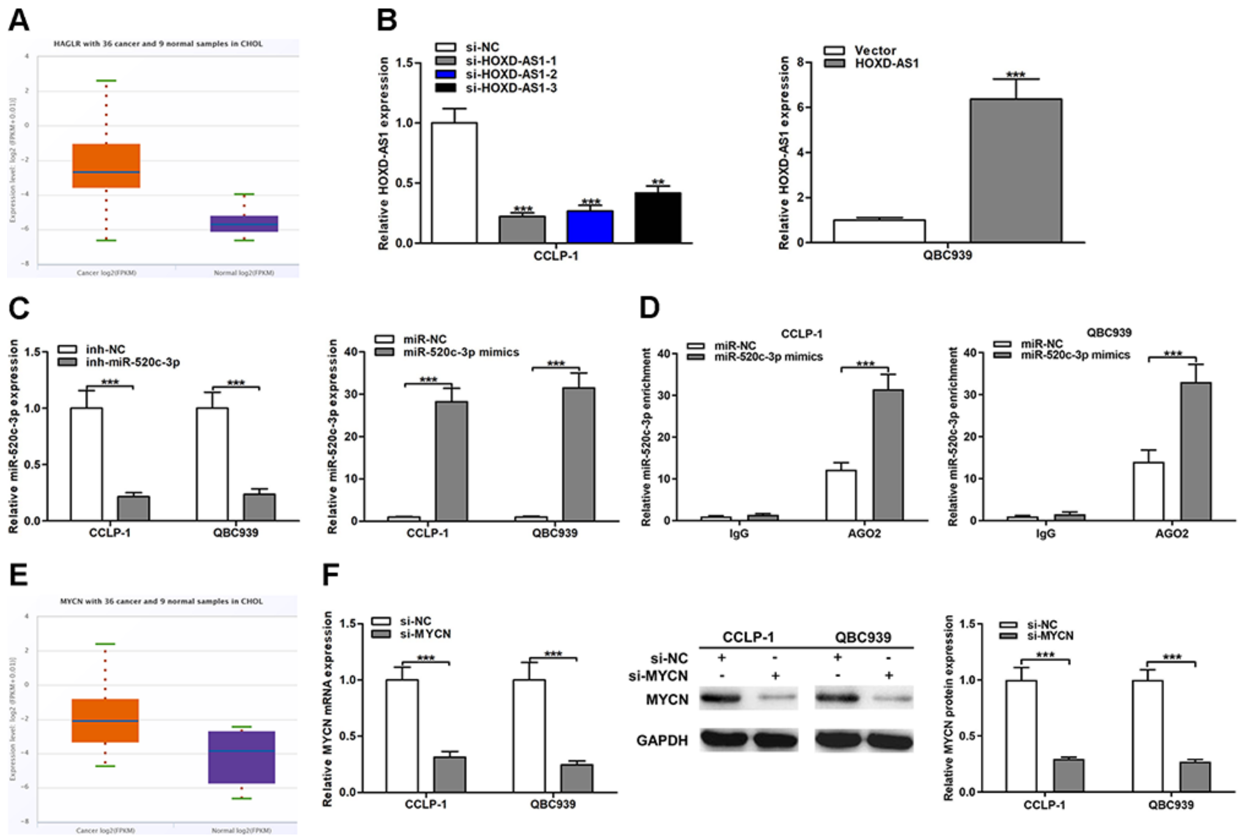

Supplementary Figure 1. Differential expression analysis in TCGA and efficiency of knockdown and amplification. (**A**) Detection of HOXD-AS1 expression in CCA samples of TCGA database. (**B**) The knockdown efficiencies of si-HOXD-AS1-1, si-HOXD-AS1-2 and si-HOXD-AS1-3 as well as amplification efficiency of pcDNA3.1-HOXD-AS1 were monitored through qRT-PCR. (**C**) The knockdown and amplification efficiencies of miR-520c-3p in CCLP-1 and QBC939 cells. (**D**) The enrichment of miR-520c-3p in AGO2 RIP assays. (**E**) Detection of MYCN expression in CCA samples of TCGA database. (**F**) The knockdown efficiency of si-MYCN in CCLP-1 and QBC939 cells was analyzed by qRT-PCR and western blot. $^{***}P < 0.001$.

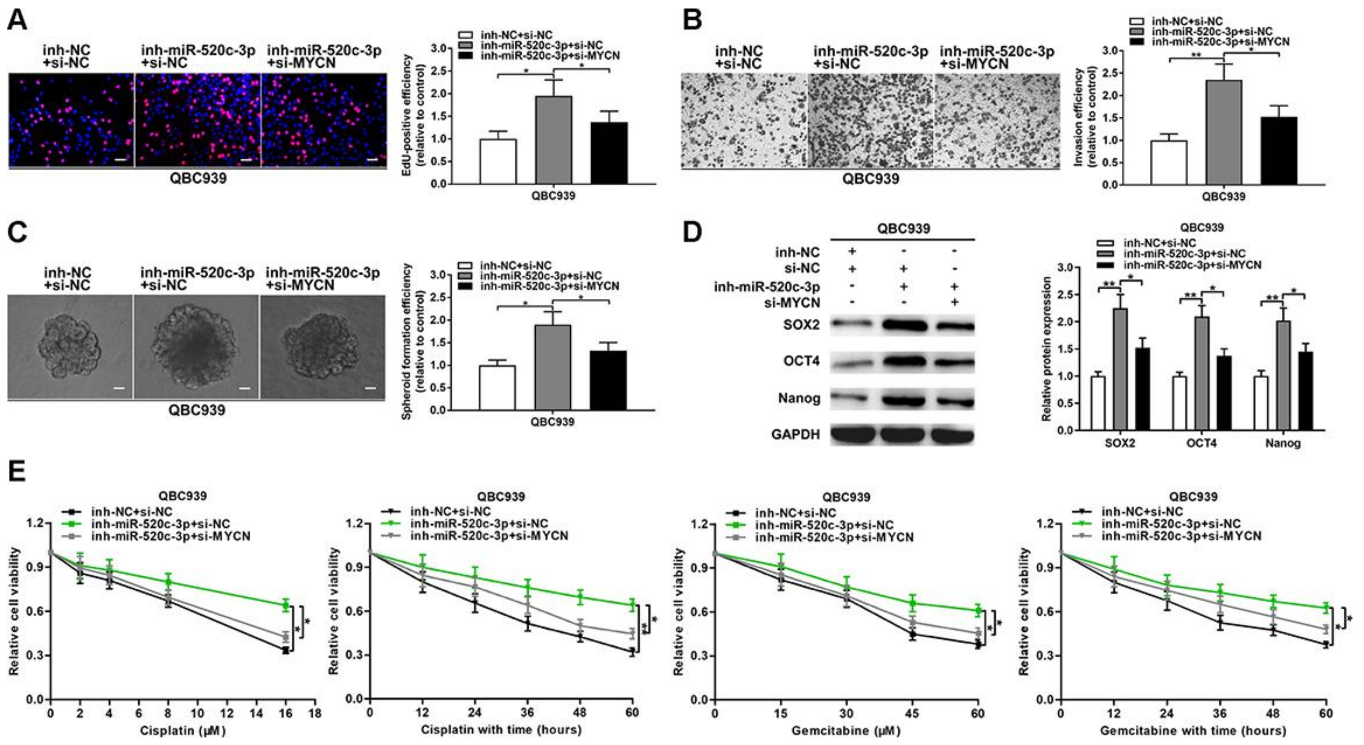

Supplementary Figure 2. MiR-520c-3p restrained CCA malignant progression by suppressing MYCN. (**A**–**E**) Rescue assays of EdU, transwell, spheroid formation, stem marker analysis and chemo-resistance confirmed that facilitation of proliferation, invasion, stemness maintenance and chemo-resistance induced by knocking down miR-520c-3p was retrieved through silencing MYCN in QBC939 cells, respectively. $^{*}P < 0.05$, $^{**}P < 0.01$.
