# Supplementary material for: SP1-induced HOXD-AS1 promotes malignant progression of cholangiocarcinoma by regulating miR-520c-3p/MYCN
Source: Aging (Albany NY). 2020 Aug 28;12(16):16304–25. doi: 10.18632/aging.103660 (PMC7485728; doi:10.18632/aging.103660)
Supplement: Supplementary Table 1 [file aging-12-103660-s001..pdf]

SUPPLEMENTARY TABLE

Supplementary Table 1. Primer sequences for qRT-PCR and sequences for siRNA.

Nucleic acids	Sequences
HOXD-AS1	Forward primer: 5'-CCTTGAAAGTGGGTAAAATGTGC-3'<br>Reverse primer: 5'-TAGTTTCCTTGTTCCTTTGTGCTGT-3'
GAPDH	Forward primer: 5'-GGGAGCCAAAAGGGTCAT-3'<br>Reverse primer: 5'-GAGTCCTTCCACGATACCAA-3'
SP1	Forward primer: 5'-GCACCTGCCCCTACTGTAAA-3'<br>Reverse primer: 5'-GTGCCTCTGTAGCTCATCCG-3'
HOXD-AS1 promoter E1 region	Forward primer: 5'-ACAGCGGTTTGGGTTTTGTG-3'<br>Reverse primer: 5'-AGAATCAAAGTCCCAGCGCA-3'
HOXD-AS1 promoter E2 region	Forward primer: 5'-ACCTCTCTATGTGCCTGAGTG-3'<br>Reverse primer: 5'-GTGTAAGAAAAGCAGAGAGCAGC-3'
U6	Forward primer: 5'-GCTTCGGCAGCACATATACTAAAAT-3'<br>Reverse primer: 5'-CGCTTCACGAATTTGCGTGTCAT-3'
miR-19b-3p	Forward primer: 5'-GTGCAAATCCATGCAAAACTGA-3'<br>Reverse primer: 5'-GTGCAGGGTCCGAGGTGCT-3'
miR-338-3p	Forward primer: 5'-TTAGTGTACCAGCCAT-3'<br>Reverse primer: 5'-GAATGCGGGAGCGAA-3'
miR-106a-5p	Forward primer: 5'-GATGCTCAAAAAGTGCTTACAGTGCA-3'<br>Reverse primer: 5'-TATGGTTGTTCTGCTCTCTGTCTC-3'
miR-665	Forward primer: 5'-GGTCTACAAAGGGAAGC-3'<br>Reverse primer: 5'-TTTGGCACTAGCACATT-3'
miR-185-5p	Forward primer: 5'-GAAGGATCCGCATGAGAGGGTGTTGGAATGC-3'<br>Reverse primer: 5'-GGAGAATTCGTGCAGGGGCAGCAGACC-3'
miR-520c-3p	Forward primer: 5'-GCCGCCAAAGTGCTTCCTTTTAG-3'<br>Reverse primer: 5'-TCGCACTGGATACGACACCCTC-3'
miR-17-5p	Forward primer: 5'-TGCAAAGTGCTTACAGTGCAG-3'<br>Reverse primer: 5'-GTGCAGGGTCCGAGGTATTC-3'
miR-421	Forward primer: 5'-CTCACTCACATCAACAGACATTAATT-3'<br>Reverse primer: 5'-TATGGTTGTTCTGCTCTCTGTGTC-3'
miR-20a-5p	Forward primer: 5'-ATGCTAAAGTGCTTATAGT-3'<br>Reverse primer: 5'-CAGTGCAGGGTCCGAGGTATTC-3'
MYCN	Forward primer: 5'-ACTGTAGCCATCCGAGGACA-3'<br>Reverse primer: 5'-CAAGCCCTGCTCCTTACCTC-3'
si-HOXD-AS1-1	5'-CAGATTCACTACTTGACTT-3'
si-HOXD-AS1-2	5'-GAAAGAAGGACCAAAGTAA-3'
si-HOXD-AS1-3	5'-GCACAAAGGAACAAGGAAA-3'
si-SP1-1	5'-UGUAGAGUCUGCCAACUGACCUGUC-3'
si-SP1-2	5'-CAGCGUUUCUGCAGCUACCUUGACU-3'
si-MYCN	5'-GAAGAAAUCGACGUGGUCA-3'
